# Differential Active Site Loop Conformations Mediate Promiscuous Activities in the Lactonase *Sso*Pox

**DOI:** 10.1371/journal.pone.0075272

**Published:** 2013-09-23

**Authors:** Julien Hiblot, Guillaume Gotthard, Mikael Elias, Eric Chabriere

**Affiliations:** 1 URMITE UMR CNRS-IRD 6236, Faculté de Médecine et de Pharmacie, Université de la Méditerranée, Marseille, France; 2 Weizmann Institute of Science, Biological Chemistry, Rehovot, Israel; University Paris Diderot-Paris 7, France

## Abstract

Enzymes are proficient catalysts that enable fast rates of Michaelis-complex formation, the chemical step and products release. These different steps may require different conformational states of the active site that have distinct binding properties. Moreover, the conformational flexibility of the active site mediates alternative, promiscuous functions. Here we focused on the lactonase *Sso*Pox from *Sulfolobus solfataricus*. *Sso*Pox is a native lactonase endowed with promiscuous phosphotriesterase activity. We identified a position in the active site loop (W263) that governs its flexibility, and thereby affects the substrate specificity of the enzyme. We isolated two different sets of substitutions at position 263 that induce two distinct conformational sampling of the active loop and characterized the structural and kinetic effects of these substitutions. These sets of mutations selectively and distinctly mediate the improvement of the promiscuous phosphotriesterase and oxo-lactonase activities of *Sso*Pox by increasing active-site loop flexibility. These observations corroborate the idea that conformational diversity governs enzymatic promiscuity and is a key feature of protein evolvability.

## Introduction

Enzymes are considered as highly efficient catalysts, so substrate recognition has long been referred to as the key-lock model with the simple view of “one sequence-one structure-one function” [1] which was subsequently refined by the induce-fit model [2]. Indeed, the protein flexibility indicates that proteins exist as a sampling of similar conformations with discrete energy levels [3,4]. Protein dynamics are essential for protein function and define its conformational landscape. Dynamics enable fast rates of Michaelis-complex formation and products release [4]. In certain cases, the transition step between conformations is the rate limiting step of enzyme catalysis [5]. Moreover, the structural diversity linked to protein flexibility constitutes a foundation of protein evolvability [4]. Indeed, the sampling of enzyme conformations allows the accommodation of different substrates within the same active site or the existence of promiscuous activities [5]. Under selective pressure, conformations allowing promiscuous binding/activities can be selected by evolution. Promiscuity is thus considered to be one of the potential engines of enzyme evolution [1,4,6-8]. Laboratory evolution can successfully exploit promiscuous activity to generate highly efficient and specialized enzymes [9,10]. However, the molecular mechanisms underlining these specializations are still under investigation.

**Figure 1:**
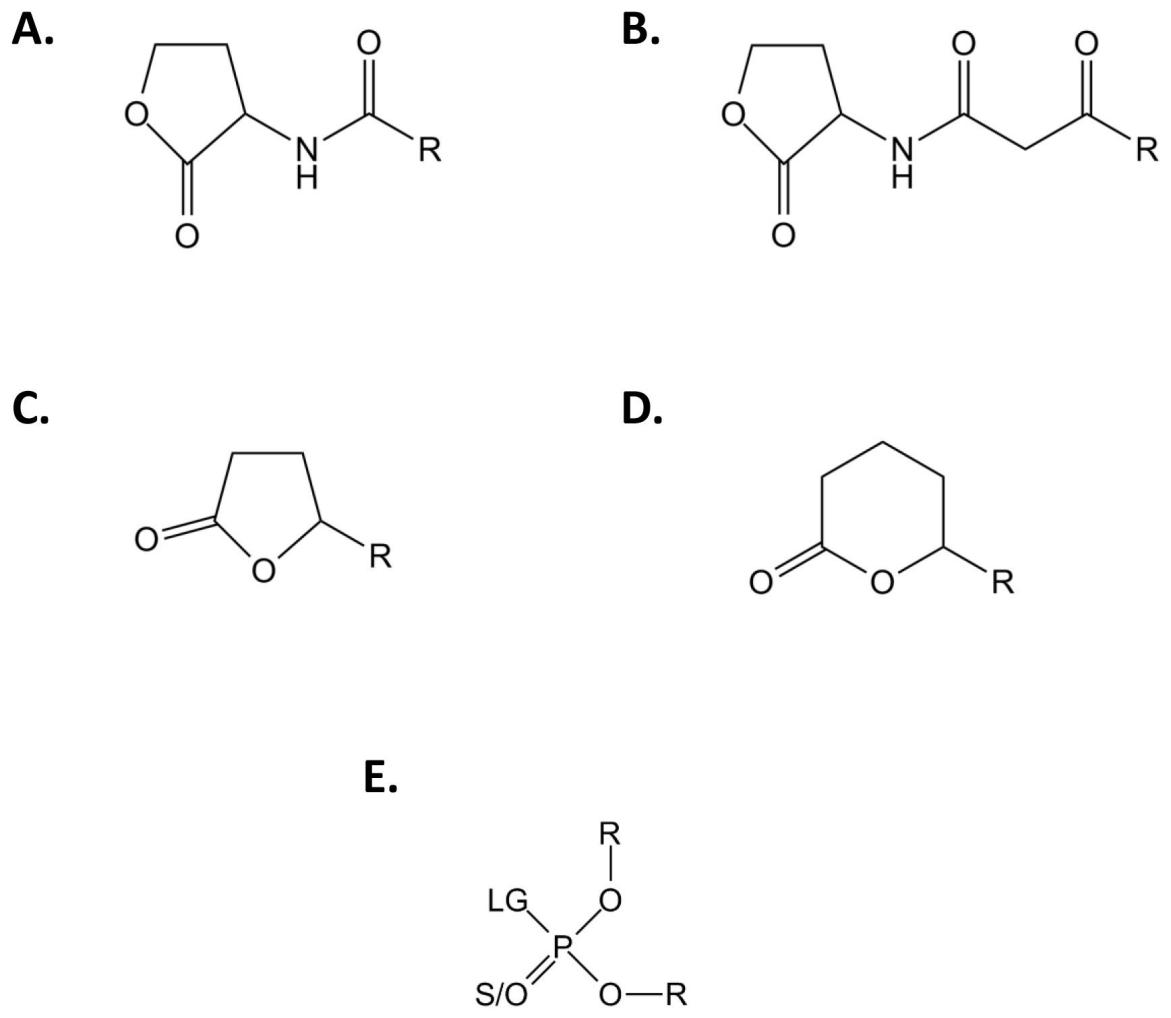
Generic chemical structure of *Sso*Pox substrates. Chemical structures of (A) Acyl-Homoserine Lactones, (B) 3-oxo-Acyl-Homoserine Lactones, (C) γ-lactones, (D) δ-lactones and (E) phosphotriesters are presented. For AHLs and γ/δ-lactones, R corresponds to the different size of the acyl chain. For phosphotriesters, R corresponds to the different nature of substituents; LG corresponds to the leaving group which can be F, S-R, O-R or CN. The terminal substituent could be a S atom if the molecule is a thiono-phosphotriester or an O atom if the molecule is an oxono-phosphotriester.

With the aim of understanding the molecular origin of enzymatic promiscuity, we focused on the Phosphotriesterase-Like Lactonases (PLLs) family. PLLs are natural lactonases endowed with promiscuous phosphotriesterase activity [6,11,12]. Isolated from bacterial [11,13-16] or archaeal [17-20] organisms, their physiological function might be related to the bacterial communication system (or *quorum* sensing) by hydrolyzing the *N*-Acyl-Homoserine-Lactones (AHLs; Figure 1AB & S1) [21,22], a *quorum* sensing molecular mediator. Other lactones (oxo-lactones; Figure 1CD & S1) are substrates for these enzymes [23], due to a coincidental binding in their active site [19]. Moreover, PLLs have long been confounded with structurally closely related bacterial phosphotriesterases (PTEs) [11], which hydrolyze paraoxon with a catalytic constant near the physical limit (i.e., k_cat_/K_M_ ~ 10^8^ M^-1^s^-1^) [24,25]. PTEs have rapidly evolved from PLLs to very efficiently hydrolyze phosphotriesters (Figure **1E & S1**), a promiscuous activity of PLLs [6,12].

PTEs and PLLs belong to the amidohydrolase superfamily and, exhibit a (α/β)_8_-barrel fold (the so-called TIM-barrel) [24,26]. This fold consists of 8 β-sheets flanked by 8 α-helixes with the catalytic center localized at the C-terminus of the barrel. The catalytic center contained two metal cations coordinated by four histidines, an aspartic acid and a carboxylated lysine residue. These metals activate a bridging water molecule, which, *via* a nucleophilic attack onto the reactive center, allows the substrate hydrolysis. The substrate specificity is mainly governed by variations in the connecting loops of the barrel. Indeed, the major structural differences between PTEs and PLLs reside in the active site loop size and conformation [27]. A structural analysis of *Sso*Pox, a hyperthermostable counterpart of the PLL family from *Sulfolobus solfataricus* [17,27,28], has revealed that loop 7 is shorter in *Sso*Pox than in PTEs; linking the active site to the loop 8, which forms a hydrophobic channel that accommodates AHLs aliphatic chain [22]. The residue W263, located in this cavity, plays a central role in substrate binding in the *Sso*Pox active site. Indeed, W263 is located at the start of loop 8, involved in the dimer interface of the enzyme and positions the lactone cycle of the substrate onto the bi-metallic catalytic center [22]. In this study, we deciphered position 263 contributions to the protein stability, enzymatic activity and promiscuity. We exhaustively mutated this position and selected the most improved variants for both phosphotriesterase and lactonase activities. By cross-analyzing the enzymatic, biochemical and structural properties of the selected variants, we isolated two distinct groups of variants and we propose structural features that may underline these improvements.

## Results

### Lactonase activity characterization of wild-type SsoPox

PLLs are subdivided into two different subfamilies based on their structures and catalytic preferences: the PLL-A (natural AHLases) and the PLL-B (natural oxo-lactonases) [12]. If lactonases such as PPH and AhlA [11] (PLL-A) exhibit equivalent AHLases and oxo-lactonase activities, *Dr*OPH [29] and *Gk*L [15] (PLL-B) comprise exclusive oxo-lactonases. The lactonase activity of *Sso*Pox, belonging to PLL-A, was assayed with various lactone substrates i.e., AHLs, δ-lactones, γ-lactones with various acyl chain lengths and other lactones (Table **1**). *Sso*Pox is an efficient enzyme against the various lactones, with catalytic efficiencies in the range of 10^4^ M^-1^s^-1^ for nonanoic-δ-latones (r) (6 atoms ring-size), 3-oxo-C10-AHLs (l) and γ-hepetanolide (r) (5 atoms ring-size) (Table **1**). As previously observed for the closely related *Sis*Lac PLL [19], *Sso*Pox exhibits an acyl chain length dependency, preferring AHLs with 8 to 10 carbon aliphatic chains and oxo-lactones with 4 to 6 carbon aliphatic chains. This feature reveals distinct accommodation modes of AHLs and oxo-lactones in the *Sso*Pox active site.

**Table 1:** *Sso*Pox lactonase activity characterization.

**Substrate**			**wild-type *Sso*Pox**			
	**Name**	**Additive**	**k_cat_ (s^-1^**)	**K_M_ (µM**)	**K_I_ (µM**)	**k_cat_/K_M_ (s** ^**-**1^ **M^-1^**)
**AHLs**	**C4-AHL (r) (V**)	-	ND	ND	ND	11.62 ± 0.72
	**C6-AHL (r) (VI**)	-	0.36 ± 0.01	459 ± 49	-	7.84 (±0.87)x10^2^
	**C8-AHL (r) (VII**)	-	1.00 ± 0.04	145 ± 26	-	6.90 (±1.27)x10^3^
	**C12-AHL (r) (VIII**)	-	1.70 ± 0.31	345 ± 90	-	4.93 (±1.56)x10^2^
**3-oxo-AHLs**	**3-oxo-C6-AHL (l**)** (IX**)	-	0.083 ± 0.003	558 ± 68	-	1.49 (±0.20)x10^2^
	**3-oxo-C6-AHL (r) (IX**)	-	0.041 ± 0.005	592 ± 176	-	6.87 (±2.20)x10^1^
	**3-oxo-C8-AHL (*l***)** (X**)	-	0.54 ± 0.02	123 ± 22	-	4.39 (±0.80)x10^3^
	**3-oxo-C8-AHL (r) (X**)	-	0.42 ± 0.02	256 ± 55	-	1.63 (±0.36)x10^3^
	**3-oxo-C10-AHL (l**)** (XI**)	-	4.52 ± 0.10	143 ± 15	-	3.16 (±0.33)x10^4^
		+ 0.1% SDS	ND	ND	-	1.96 (±0.04)x10^2^
		+ 0.01% SDS	0.75 ±0.03	243±43	-	3.09 (±0.56)x10^3^
	**3-oxo-C12-AHL (l**)** (XII**)	-	1.01 ± 0.13	456 ± 128	-	2.22 (±0.68)x10^3^
**γ-lactones**	**γ-butyrolactone (XIII**)	-	ND	ND	ND	1.20 (±0.12)x10^3^
	**γ-heptanolide (r) (XIV**)	-	2.92 ± 0.08	166 ± 21	-	1.76 (±0.23)x10^4^
	**Nonanoic-γ-lactone (r) (XV**)	-	5.54 ± 0.57	2 943 ± 436	-	1.88 (±0.34)x10^3^
	**Undecanoic-γ-lactone (r) (XVI**)	-	4.95 ± 0.26	2 099 ± 230	-	2.36 (±0.29)x10^3^
		+ 0.1% SDS	2.23 ± 0.47	1 250 ± 361	1 470 ± 440	1.78 (±0.64)x10^3^
		+ 0.01% SDS	0.46 ± 0.01	94 ± 10	-	4.89 (± 0.53)x10^3^
	**Dodecanoic-γ-lactone (r) (XVII**)	-	2.72 ± 0.13	1 220 ± 144	-	2.23 (± 0.28)x10^3^
**δ-lactones**	**δ-valerolactone (XVIII**)	-	ND	ND	ND	ND
	**Nonanoic-δ-lactone (r) (XIX**)	-	15.32 ± 0.52	359 ± 63	-	4.27 (±0.77)x10^4^
	**Undecanoic-δ-lactone (r) (XX**)	-	7.38 ± 0.28	94 ± 18	-	7.86 (±1.53)x10^4^
	**Dodecanoic-δ-lactone (r) (XXI**)	-	12.65 ± 0.44	1 678 ± 133	-	7.54 (±0.65)x10^3^
**Others**	**ε-caprolactone (XXII**)	-	4.45 ± 0.08	234 ± 18	-	1.90 (±0.15)x10^4^
	**Dihydrocoumarin (XXIII**)	-	7.32 ± 1.25	1 376 ± 455	-	5.32 (±1.98)x10^3^

Roman numbers indicate the chemical structures of indicated molecules presented in Figure **S1**. *r* corresponds to racemic solution and *l* at the pure levorotatory enantiomer. Data obtained with cobalt as cofactor. ND corresponds to an undetermined value. When V_max_ could not be reached, the linear part of the MM plot was fitted to a linear regression and corresponded to the catalytic efficiency. *Sso*Pox clearly preferred the levorotatory enantiomer of the AHLs; kinetics performed for the racemic mix resulted in characterization for the levorotatory enantiomer.

PLLs are believed to be natural AHL lactonases, possibly involved in *quorum* quenching [12,22]. Interestingly, PLLs enzymes, such as *Sso*Pox, hydrolyze a broad spectrum of different lactones. Noteworthy, anionic detergents, which probably increase protein flexibility, strongly stimulate *Sso*Pox promiscuous phosphotriesterase activity (up to 33-fold) [30,31], whereas they have an opposite effect on lactonase activities. In the presence of 0.1% SDS, the enzyme activity towards the best AHL substrate (3-oxo-C10 AHL (*l*)) is severely compromised (161-fold decrease), and, the activity towards undecanoic-γ-lactone (r) is mildly affected (1.3-fold decrease) (Table **1**). Lower SDS concentrations (0.01%) yield to milder but similar effects: the catalytic efficiency against 3-oxo-C10 AHL (l) (10-fold) is decreaed, and undecanoic-γ-lactone (r) hydrolysis is increased (2-fold) (Table **1**). Alterations in substrate specificity induced by detergent were previously observed in enzymes [32], and were mainly attributed to detergent-induced protein flexibility that enlarged the protein conformational landscape [33]. As for *Sso*Pox, detergent-induced flexibility could promotes the increase of promiscuous phosphotriesterase activity but dramatically compromised the AHLase activity, possibly by altering the specific loop conformational sampling required for the proper binding / hydrolysis of AHLs. This result is consistent with AHLs as the native substrates of *Sso*Pox.

Additionally, we investigated the enantiospecificity of *Sso*Pox towards AHLs by using racemic and pure levorotatory 3-oxo-C8 AHLs (Figure **S2**). The levorotatory enantiomer (Specific Activity for the levorotatory enantiomer [i.e., SA_(l)_] = 5.5 (±0.5)x10^-1^ s^-1^) is hydrolyzed at approximately 2-fold higher velocity than the racemic one (SA_1(r)_ = 3.69 (±0.01)x10^-1^ s^-1^). Moreover, racemic AHLs hydrolysis by wild-type *Sso*Pox exhibits a biphasic curve. The second recorded velocity (SA_2(r)_ = 1.47 (±0.02)x10^-2^ s^-1^) presents an approximately 20-fold lower hydrolysis of the dextrorotatory enantiomer highlighting the wild-type *Sso*Pox preference for levorotatory AHLs. The catalytic efficiencies obtained for racemic 3-oxo-C8 AHLs and 3-oxo-C6 AHLs are approximately half of pure levorotatory AHLs. These results confirm the enantiopreference of wild-type *Sso*Pox (Table **1**) for naturally used molecules in bacterial *quorum* sensing [34].

### Isolation of W263 variants with improved phosphotriesterase and lactonase activities

#### Phosphotriesterase activity screening

**Figure 2:**
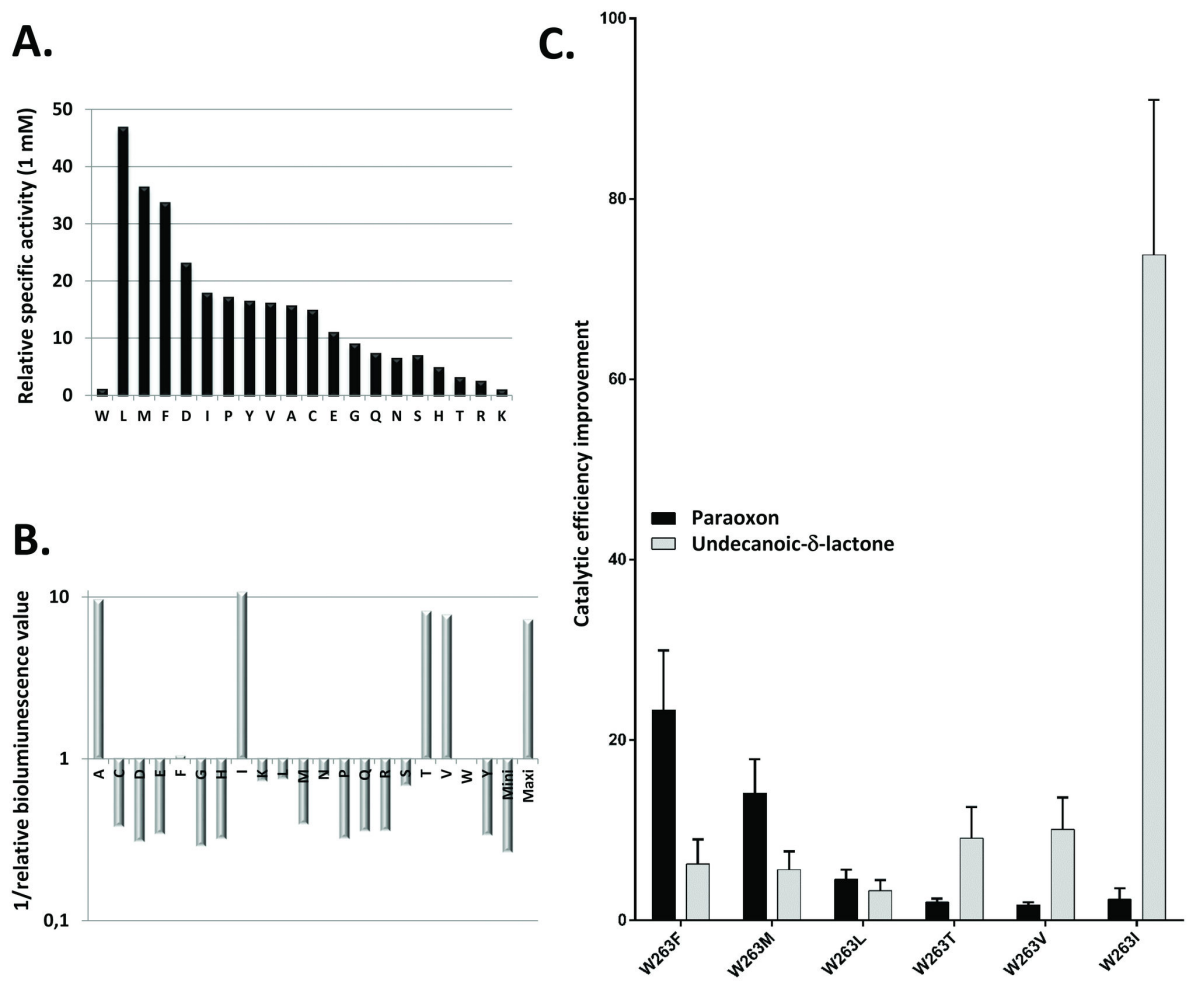
*Sso*Pox W263 saturation site screening and characterization. **A**. Relative phosphotriesterase activities of W263 saturation site variants are screened with 1 mM of paraoxon substrate. **B**. Relative lactonase activity of W263 saturation site variants screened for 3-oxo-C12 AHL hydrolysis (see methods for more details). **C**. Catalytic efficiency comparisons between best selected variants. For screening experiments (A-B.), partially purified enzymes (> 70%) were used.

All selected variants of the saturation site of position 263 have been produced and partially purified in a heating step (see methods). This method might induce a bias resulting from the different expression of variants, but has the merit of enabling fast selection and comparison of the activities of the various expressed proteins. The ability of each variant to hydrolyze paraoxon has been evaluated with 1 mM (Figure **2A**) and 100 µM (Figure **S3A**). Compared to wild-type *Sso*Pox, variants W263L, W263M and W263F are the most efficient at improving specific activities, ranging from 30-50 and 20-35-fold at 1 mM and 100 µM, respectively. These variants constitute the *Phosphotriesterase Selected Variants* group (*PteSV*). Notably, all 19 substitutions of position 263 increase paraoxonase activity (Figure **2A**). These variants are also the most proficient at hydrolyzing CMP-coumarin (an organophosphate nerve agent analogue; Figure **S1-II**) with specific activity improvements ranging from 4- to 11-fold compared to wild-type enzyme (Figure **S3B**).

#### Lactonase activity screening

The lactonase activity of each variant has been screened using a genetically modified *Pseudomonas aeruginosa*-based bioluminescence screening method (Figure **S4**). Variants W263I, W263T and W263V were selected (Figure **2B**) and constitute the *Lactonase Selected* Variant group (*LacSV*).

### Phosphotriesterase kinetic characterization of selected variants

Catalytic efficiencies of paraoxon hydrolysis have been determined for the selected variants (*PteSV* group) (Table **2**). Variant W263F is the most proficient (k_cat_/K_M_ = 1.21 (±0.26)x10^4^ M^-1^s^-1^), followed by W263M and W263L with increased efficiencies of 23.3, 14.1 and 4.6-fold, respectively, as compared to wild-type enzyme. In contrast, variants isolated for their improved lactonase activity (*LacSV*) all exhibit mild catalytic improvement compared to wild-type enzyme ranging from 2.3-1.7 times, respectively, for W263I-W263V (Table **2**; Figure **2C** **& S5AB**).

**Table 2:** Enzymatic characterization of wild-type *Sso*Pox and its variants.

	*Sso*Pox variant	k_cat_ (s^-1^)	K_M_ (µM)	K_I_ (µM)	k_cat_/K_M_ (M^-1^s^-1^)	Enhancement/wt
Paraoxon (I)	*wt* *	(1.26 ± 0.13)x10^1^	24250 ± 3716	-	5.19 (±0.95)x10^2^	1
	W263F	8.47 ± 0.53	700 ± 146	-	1.21 (±0.26)x10^4^	23.3 ± 6.6
	W263M	6.82 ± 0.57	931 ± 163	-	7.33 (±1.42)x10^3^	14.1 ± 3.8
	W263L	ND	ND	-	2.37 (±0.33)x10^3^	4.6 ± 1.0
	W263I	ND	ND	-	1.21 (±0.06)x10^3^	2.3 ± 0.4
	W263V	ND	ND	-	8.83 (±0.3)x10^2^	1.7 ± 0.3
	W263T	ND	ND	-	1.06 (±0.03)x10^3^	2.0 ± 0.4
3-oxo-C12 AHL (l) (XII)	*wt*	1.01 ± 0.13	456 ± 128	-	2.22 (±0.68)x10^3^	1
	W263F	0.41 ± 0.02	146 ± 33	-	2.81 (±0.65)x10^3^	1.3 ± 0.5
	W263M	ND	ND	-	ND	ND
	W263L	ND	ND	-	ND	ND
	W263I	1.80 ± 0.05	17.8 ± 4.9	-	1.01 (±0.28)x10^5^	45.5 ± 18.8
	W263V	3.00 ± 0.07	24.7 ± 5.2	-	1.21 (±0.26)x10^5^	54.5 ± 20.4
	W263T	6.44 ± 0.22	137 ± 19	-	4.70 (±0.67)x10^4^	21.2 ± 7.2
3-oxo-C10 AHL (l) (XI)	*wt*	4.52 ± 0.10	143 ± 15	-	3.16 (±0.40)x10^4^	1
	W263F	3.96 ± 0.18	288 ± 56	-	1.38 (±0.28)x10^4^	4.4 (±1.0)x10^-1^
	W263M	ND	ND	-	ND	0
	W263L	ND	ND	-	ND	0
	W263I	(6.00 ± 0.90)x10^-1^	1605 ± 443	-	3.74 (±1.17)x10^2^	1.2 (±0.4)x10^-2^
	W263V	(1.90 ± 0.09)x10^-1^	1346 ± 298	-	1.41 (±0.32)x10^2^	4.5 (±1.2)10^-3^
	W263T	(1.07 ± 0.16)x10^-1^	1000 ± 343	-	1.06 (±0.40)x10^2^	3.4 (±1.3)10^-3^
Undecanoic-δ-lactone (r) (XX)	*wt*	7.38 ± 0.28	94 ± 18	-	7.86 (±1.53)x10^4^	1
	W263F	(6.65 ± 0.32)x10^1^	135.2 ± 52.8	-	4.92 (±1.93)x10^5^	6.3 ± 2.7
	W263M	(7.12 ± 0.66)x10^1^	161 ± 47	7 400 ± 2 475	4.42 (±1.35)x10^5^	5.6 ± 2.0
	W263L	(5.68 ± 0.58)x10^1^	219 ± 62	4 253 ± 1 152	2.59 (±0.78)x10^5^	3.3 ± 1.2
	W263I	(5.80 ± 0.74)x10^1^	<10	803 ± 213	>5.80 (±0.74)x10^6^	>73.8 ± 17.2
	W263V	(4.48 ± 0.50)x10^1^	57 ± 16	789 ± 186	7.92 (±2.34)x10^5^	10.1 ± 3.6
	W263T	(9.33 ±0.80)x10^1^	130 ± 41	3047 ± 576	7.17 (±2.34)x10^5^	9.1 ± 3.5
Undecanoic-γ-lactone (r) (XVI)	*wt*	4.95 ± 0.26	2 099 ± 230	-	2.36 (±0.38)x10^3^	1
	W263F	4.63 ± 0.27	373 ± 111	-	1.24 (±0.38)x10^4^	5.3 ± 1.8
	W263M	4.25 ± 0.22	334 ± 61	-	1.27 (±0.24)x10^4^	5.4 ± 1.3
	W263L	3.92 ± 0.17	371.8 ± 69.2	-	1.05 (±0.20)x10^4^	4.4 ± 1.1
	W263I	1.94 ± 0.08	361 ± 47	-	5.37 (±0.73)x10^3^	2.3 ± 0.5
	W263V	5.64 ± 0.53	1 760 ± 404	-	3.20 (±0.80)x10^3^	1.4 ± 0.4
	W263T	4.55 ± 0.10	13.0 ± 4.2	-	3.49 (±1.13)x10^5^	147.9 ± 53.5

Roman numbers indicate the chemical structures of indicated molecules presented in Figure **S1**. * Catalytic parameters are from Hiblot et al. (2012). ND corresponds to an undetermined value. When V_max_ could not be reached, the linear part of the MM plot was fitted to a linear regression and correspond to the catalytic efficiency.

The most active variant W263F has been further assayed using other organophosphates as substrates (Table **3**). The catalytic parameters obtained for CMP-coumarin (k_cat_/K_M_ = 8.23 (±1.04)x10^4^ M^-1^s^-1^), IMP-coumarin (k_cat_/K_M_ = 8.85 (±2.99)x10^4^ M^-1^s^-1^) and PinP-coumarin (k_cat_/K_M_ = 7.08 (±2.58)x10^3^ M^-1^s^-1^) hydrolysis enhanced catalytic efficiency 10- and 60-fold compared to the wild-type enzyme for which no activity was reported for PinP-coumarin [30]. The variant W263F was also assayed in the presence of SDS, because anionic detergents have been shown to increase the phosphotriesterase activity of wild-type *Sso*Pox [30,31]. Interestingly, SDS has lower influence on the kinetic parameters of variant W263F against paraoxon and CMP-coumarin (with a catalytic efficiency increase ranging from 1.7 to 6.1-fold) than that observed for wild-type *Sso*Pox (ranging from 6.4 to 12.4-fold) [30]. Activity improvement by SDS is caused by a global increase of the protein flexibility [30]. Thus, the W263F mutation might then mimic the SDS effect, by bringing local flexibility into the active site.

**Table 3:** Phosphotriesterase activity comparison between wild-type and W263F *Sso*Pox.

**Substrate**		*Sso*Pox wt*			***Sso*Pox W263F**		
**Name**	**Additive**	**k_cat_ (s^-1^**)	**k_M_ (µM**)	**k_cat_/k_M_ (s** ^**-**1^ **M^-1^**)	**k_cat_ (s^-1^**)	**k_M_ (µM**)	**k_cat_/k_M_ (s** ^**-**1^ **M^-1^**)
**Paraoxon (I**)	-	12.59 ± 1.26	24 250 ± 3 716	5.19 (±0.95)x10^2^	8.47 ± 0.53	700 ± 146	1.21 (±0.26)x10^4^
	+ SDS 0.1%	40.72 ± 7.70	12 340 ± 3 625	3.30 (±1.15)x10^3^	117.7 ± 6.0	2 462 ± 302	4.78 (±0.64)x10^4^
	+ SDS 0.01%	24.59 ± 1.77	3 832 ± 626	6.42 (±1.15)x10^3^	85.85 ± 4.85	1 168 ± 194	7.35 (±1.29)x10^4^
**CMP-coumarin (II**)	-	ND	ND	8.13 (±0.08)x10^3^	9.41 ± 0.38	114.3 ± 13.7	8.23 (±1.04)x10^4^
	+ SDS 0.01%	25.47 ± 0.42	137.0 ± 7.0	1.86 (±0.10)x10^5^	8.64 ± 0.39	60.0 ± 9.5	1.44 (±0.24)x10^5^
**IMP-coumarin (III**)	-	ND	ND	1.67 (±0.04)x10^3^	8.39 ± 1.63	94.8 ± 26.2	8.85 (±2.99)x10^4^
**PinP-coumarin (IV**)	-	ND	ND	ND	0.11 ±0.02	16.1 ± 5.49	7.08 (±2.58)x10^3^

Roman numbers indicate the chemical structures of indicated molecules presented in Figure **S1**. * Catalytic parameters are from Hiblot et al. (2012). ND corresponds to an undetermined value. When V_max_ could not be reached, the linear part of the MM plot was fitted to a linear regression and correspond to the catalytic efficiency.

### Lactonase kinetic characterization of selected variants

Selected variants have been characterized with the best, possibly natural, substrate of wild-type *Sso*Pox (3-oxo-C10 AHL), the worse AHL substrate (3-oxo-C12) and promiscuous oxo-lactones with long aliphatic chains (undecanoic-δ-lactones and undecanoic-γ-lactones) (Table **2**). The kinetic experiments reveal a mild decrease in the 3-oxo-C10 AHLase activity for all variants (ranging from 2- to 3-fold for the W263F variant to undetectable activities for W263L-M variants) (Figure **S5A**). On the contrary, the moderate 3-oxo-C12 AHLase activity of wild-type *Sso*Pox was improved by 1.3- to 55-fold (W263F and W263V, respectively), but no activity was detected for variants W263L-M (Figure **S5A**). Regarding the other lactones, all selected variants possess higher catalytic efficiencies than the wild-type enzyme, ranging from 1.4- (W263V) to 148-fold for undecanoic-γ-lactone (W263T) (Figure **S5**) and from 3.3- (W263L) to > 74-fold for undecanoic-δ-lactones (W263I) (Figure **2C** **& Figure S5AC**). Notably, contrary to the wild-type enzyme, all selected variants (with the exception of W263F) exhibit a substrate inhibition for undecanoic-δ-lactones with K_I_ values ranging between 789 ± 186 µM and 7 400 ± 2 475 µM, respectively, for the W263V and W263M variants. This substrate inhibition has not been observed for undecanoic-γ-lactones and AHLs. Because these mutants dramatically increase the δ- and γ-lactonase activities (up to 148-fold), but have a less important effect on AHLs (up to 55-fold improvement for 3-oxo-C12 AHL) or even decrease this activity (with 3-oxo-C10 AHL), these classes of lactones may utilize a different binding mode to the active site and/or a different active site conformation.

### Thermal stability and thermophilicity of *SsoPox* variants

**Figure 3:**
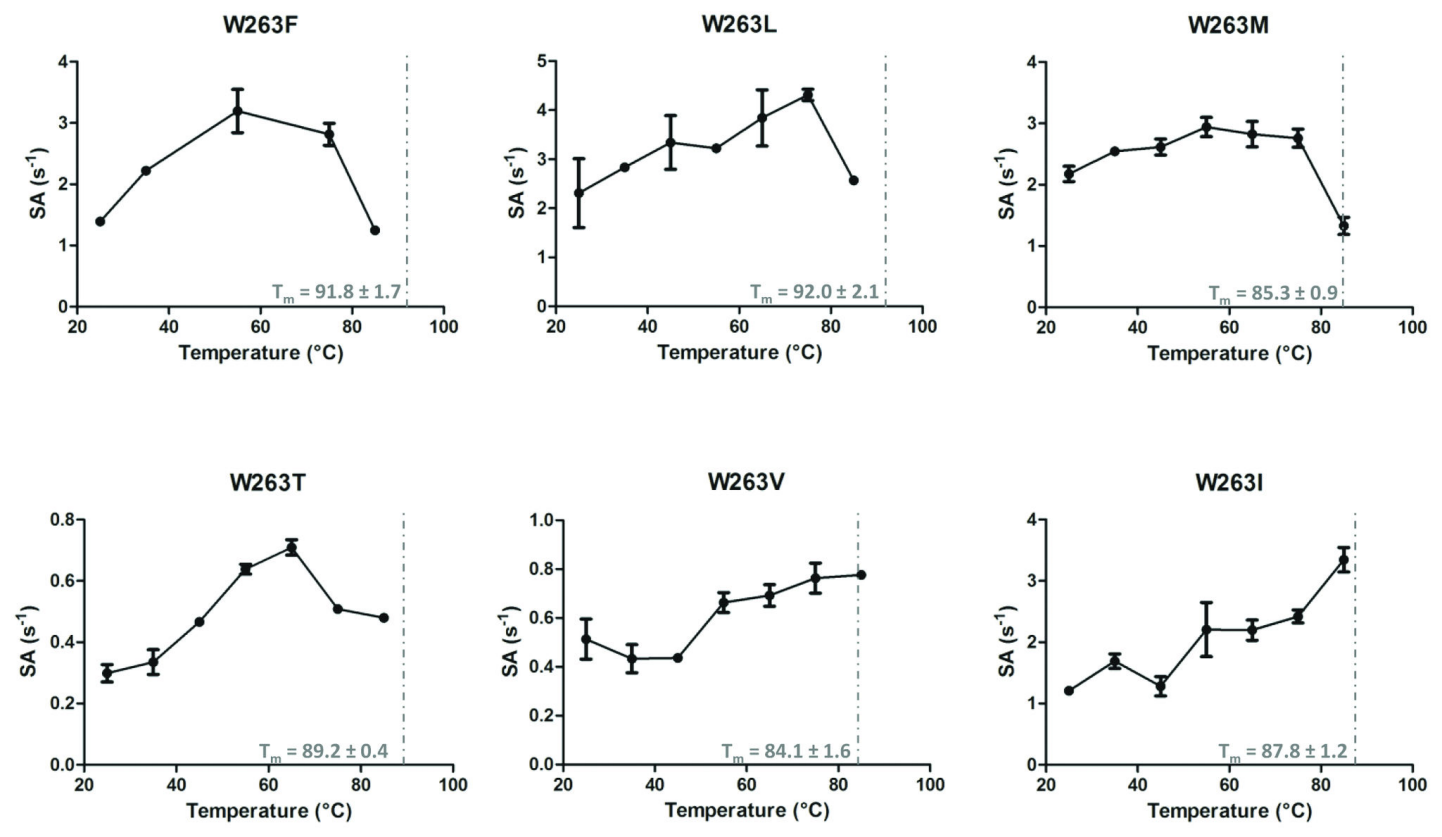
Thermoresistance and thermophilicity of *Sso*Pox variants. Specific activities (in mol. mol^-1^.s^-1^) for 50 µM of paraoxon are represented at several temperatures ranging from 25 to 85 °C for each selected variants. The T_m_ value is indicated by a grey dashed line. The wild-type enzyme has a reported T_m_ of 106 °C [28], and its paraoxonase activity continuously increases with temperature [17].

*Sso*Pox is an extremely thermostable enzyme (melting temperatures (T_m_) = 106 °C) [27]. We here show that substitutions at position 263 are destabilizing (Figure **3**; Table **S1**). Indeed, all variants exhibit a lower T_m_ compared to the wild-type enzyme (between 92.0 ± 2.1 °C for *Sso*Pox-W263L to 84.1 ± 1.6 °C for *Sso*Pox-W263V). In addition to *Sso*Pox-W263M, *PteSV* variants possess a higher thermal stability than *LacSV* variants. Contrary to the wild-type enzyme that did not lose any paraoxonase activity with an increase in temperature [17], the selected variants possess different profiles (Figure **3**). Interestingly, the loss of paraoxonase activity for the variants *PteSV* occurs before the loss of structure (corresponding to the T_m_ value). On the contrary, the paraoxonase activity of *LacSV* variants exhibit a behavior that is similar to that of the wild-type enzyme, i.e., the activity keeps increasing with temperature (or remains higher than ambient temperature activity for W236T around its T_m_). The temperature-induced behaviors of *LacSV* and *PteSV* confirm their respective distinct behaviors observed in enzyme kinetics.

### Structural characterization

In order to understand the structural consequences of variations at position 263, the crystal structures of all selected variants have been solved. No major differences in the wild-type enzyme structure are observed. Position 263 is located at the dimer interface [22]; and it modulates the relative orientation of its contacting residues, F104, in the second monomer (Figure **S6A**). Consequently, substitutions of W263 affect the relative orientation of both monomers; for each mutant, the monomers are closer to each other compared to the wild-type enzyme (with the exception of W263L). The dimer reorientation yields significant movements that, ranging from 3.4 Å (W263T) to 4.7 Å (W263I) compared to wild-type *Sso*Pox (Figure **S6C**; Table **S1**).

The active sites of variant structures superimpose well with the wild-type enzyme structure (**Figure S6AB**). However, the different residues selected at position 263 modulate the size of the active site cavity, which is increased as compared to wild-type enzyme. The active site cavity is globally larger for *LacSV* than for *PteSV* (Figure **S7**). The HTL-bound structure (Figure **S8A**) of the lactonase variant W263I exhibits the same binding mode than that observed in the wild-type *Sso*Pox structure [22]: both structures are well superimposed (Figure **S8B**). This feature suggests that the observed catalytic enhancements are related to substrate binding, rather than to catalysis improvement. The loss of the interaction of the lactone ring with W263 is compensated by an interaction with a polyethylene glycol molecule that fills the cavity created by the W263I mutation (Figure **S8A**).

**Figure 4:**
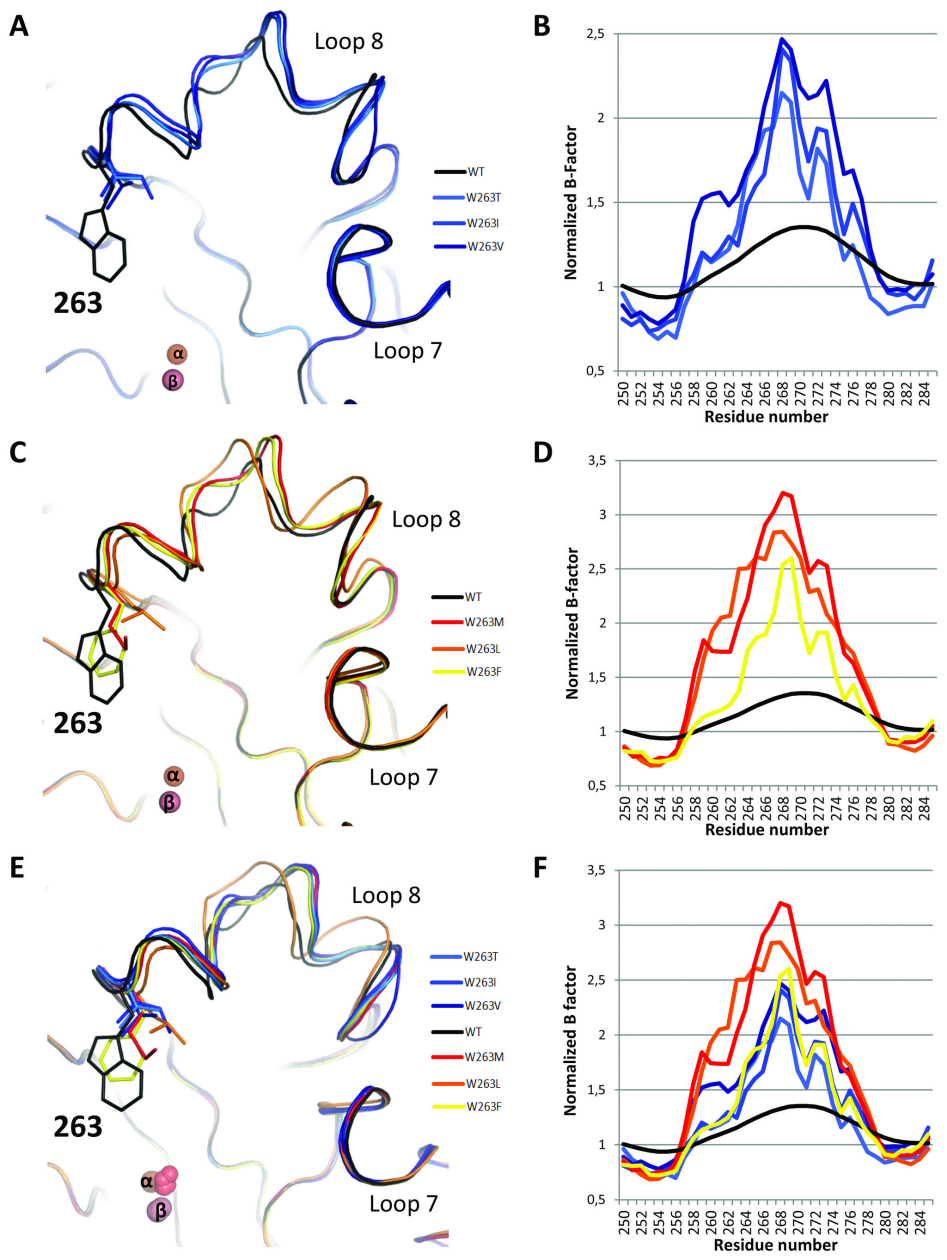
Different loop 8 conformations of W263 mutants. Backbone representation of superposition (**A**-**C**-**E**) and normalized B-factors (**B**-**D**-**F**) of *LacSV* (**A**-**B**). *PteSV* (**C**-**D**) and all selected variants (**E**-**F**) compared to wild-type *Sso*Pox in the active site region (Loop 8). Normalized B-factors are represented on the region 250-285; it corresponds to the B-factor of each residue normalized by the mean B-factor of the structure.

The main difference, albeit subtle, between all structures relate to the conformation of the active site loop 8, which carries the position 263 [22]. Indeed, all selected variants present a slightly altered loop 8 conformation compared to wild-type enzyme (**Figure 4ACE**). All variants possess loop 8 conformations that are similar (Figure **4E**) with the exception of the W263L variant (Figures 4C & **S9A**). Moreover, the normalized B-factor confirms that the substitution of W263 is highly destabilizing, because the normalized B-factor of the loop is increased in all variants, compared to the wild-type structure (**Figure 4BDF** **& S9B**). The increased flexibility (or increased normalized B-factor) is even more pronounced in *PteSV* variants than in *LacSV* variants (**Figure 4BD**). 

## Discussion

### W263 substitutions increase *SsoPox* promiscuous activities

A single mutation of W263 can significantly increase the paraoxonase activity of the enzyme (by 23-fold with the W263F substitution), the oxo-lactonase activity (by 148-fold with the W263T substitution) and the activity toward the poor AHL substrate (3-oxo-C12 AHL; by 55-fold (W263V)). The impact of this substitution is such that *Sso*Pox-W263F hydrolyzes nerve agent analogs with remarkable efficiency (k_cat_/K_M_ is 8.85 (±2.99)x10^4^ M^-1^s^-1^ against IMP-coumarin), whereas the wild-type *Sso*Pox is unable to hydrolyze this compound [19]. Noteworthy, subtle changes, such as W263L or W263I, yield to a large tradeoff in catalytic activities.

The W263 substitution seems to have similar effect as SDS on *Sso*Pox: promiscuous phosphotriesterase activity is increased in both cases. Interestingly, SDS has a small stimulatory effect on *Sso*Pox-W263F catalytic efficiency (1.7-6.1-fold increase), whereas it strongly stimulates the wild-type enzyme (with paraoxon and CMP-coumarin as substrates; 6.4- to 12.4-fold increase) [19]. Thus, the detergent induced-flexibility and substitutions of W263 may have overlapping structural effects on the enzyme. However, whereas detergent-induced flexibility has no effect on the lactonase activity, we isolated variants of W263 that exhibit increased lactonase activities (W263T/V/I). These variants exhibit dramatic improvement of their oxo-lactonase catalytic efficiencies (148-fold for variant W263T), while the activity for the best AHL substrate is compromised (3-oxo-C10 AHL; for the wild-type enzyme).

Residue W263 of *Sso*Pox is a key position that interacts with the substrate, is located in loop 8 and is involved in the dimer interface [22] (Figure **S6**). The effects of W263 substitutions on the enzyme structure are numerous, because the W263 mutation provokes a reshaping of the active site cavity (Figure **S7**) and, for some variants a slight re-orientation of the protein homodimer (Figure **S6**). These structural features may contribute to the observed variation of catalytic activities in the W263 variants, compared to the wild-type enzyme. However, the modulation of the enzyme dimer are not systematic for all variants, and therefore cannot explain the systematic effect of W263 on promiscuous activities. Additionally, our study demonstrates that all 19 possible substitutions of the W263 residue increase the *Sso*Pox paraoxonase activity. Such a global effect suggests a non-specific effect of the substitutions on the catalytic cycle, and eliminates a specific role of W263 mutants in the promiscuous substrates accommodation. Moreover, the substitutions of W263 have another effect on the enzyme: all substitutions are consistently destabilizing (Figure **3**) and dramatically increase the active site loop 8 flexibility (Figure **4**).

### A given promiscuous activity requires a given conformational subset of the active site loop

Interestingly, the selected mutations for increased phosphotriesterase and lactonase activities (*PteSV* and *LacSV*) are overall mutually exclusive and constitute two distinct groups. Regarding the *PteSV* group, while these variants exhibit higher phosphotriesterase activity, they demonstrate mild improvements for the oxo-lactones and no or wild-type-like AHLase activity (W263L-M and W263F, respectively). Conversely, the *LacSV* group dramatically increases oxo-lactonase activity and 3-oxo-C12 AHLase activities, while the phosphotriesterase activity is mildly modulated and 3-oxo-C10 AHLase is decreased. This dichotomy between the two groups of substitutions is further illustrated by different active-site loop conformational properties. The residues selected in the *LacSV* group (T, V, I) are overall smaller than those selected in the *PteSV* group (F, L, M) with the exception of isoleucine (I). Mutations W263V-T therefore yield enlarged active site cavities for the lactone variants. The analysis of the normalized B-factors of the variants clearly reveal that the substitution W263 is destabilizing (as confirmed by the T_m_ of the variants), but also that the active site loops of *PteSV* are significantly more disordered than those of *LacSV*. This feature is nicely illustrated by the thermophilicity profile of these variants. *PteSV* lose their activity before the loss of the global structure (T_m_), whereas *LacSV* do not, and exhibit a similar thermophilicity profile to that of the wild-type enzyme [17]. These data strongly suggest that the increased disorder in loop 8 caused by the *PteSV* substitutions collapses the loop as the temperature increase, whereas it does not occur with the *LacSV*.

The consequences of the W263 substitution are a decrease in the overall protein stability, a reshaping of the active-site cavity, a slight re-orientation of the enzyme homodimer, a very important increase in loop 8 flexibility and concomitantly an increase in the enzyme’s promiscuous activities. The different group of W263 substitutions yield to different conformational loop samplings that possess different structural and physical properties, and are distinct from wild-type behavior. Moreover, because the activity profiles of *LacSV* and *PteSV* show little overlap, the promiscuous activities may then require not only different loop conformations, but also different subsets of the conformational loop landscape. The enzyme would then use a given conformational subset of the active-site to process a given molecule. More precisely, this conformational subset would produce one specific loop conformation that allows the productive binding of the given substrate and another that permits an efficient release of corresponding products. The fact that all selected mutations, for both group of activities, dramatically lower the K_M_ of the enzyme is consistent with the idea that the conformational flexibility required for enhancing promiscuous activities mainly improves the substrate binding (or reduces non-productive bindings), and/or products release but not catalysis (i.e. the chemical step).

Similarly to the case of paraoxonase 1, the promiscuous activities utilize the catalytic machinery in different combinations [6-8]. This different usage of the active site enables the proper alignment of the promiscuous substrates and the catalytic residues [1,7,8]. Position W263 thus exemplifies a point mutation that can dramatically alter enzyme specificity without the complete loss of the native function (weak tradeoff) [4]. Such a mutation may then provide an evolutionary advantage to the organism, and give birth to a specialized novel enzyme after gene duplication. Flexible loops in the enzyme support the notion of fold polarity [35], whereby a part of the active site (the loop) is weakly connected to the protein scaffold and thus provides the potential, with little mutational events, for evolving new functions. 

## Material and Methods

### Screening methods

#### Sample preparation for the screening steps

A site-saturation of position W263 of *Sso*Pox was ordered to a service provider (GeneArt, Invitrogen, Germany). Each variant were checked by sequencing and stored as *Escherichia coli* DH5α cell glycerol stocks. The 20 plasmids (pET22b-*Sso*Pox-W263X [30]) have been purified from *E. coli* DH5α cells and transformed into the BL21(DE_3_)-pLysS strain for protein production. Protein production was performed in 3 mL of ZYP medium [36] (100 µg/ml ampicillin, 34 µg/ml chloramphenicol) as previously described [19,20,30]. Cells were harvested by centrifugation (3 000 ×g, 4 °C, 10 min), re-suspended in 500 µL of *lysis buffer* (50 mM HEPES pH 8, 150 mM NaCl, 0.2 mM CoCl_2_, 0.25 mg/ml lysozyme, 0.1 mM PMSF and 10 µg/ml DNAseI) and stored at -80°C. Suspended frozen cells were thawed and disrupted by three steps of 45 seconds of sonication (Ultrasonic processor xl; power 5). Cell debris were removed by centrifugation (13 000 ×g, 25 °C, 30 min). Partial purification of the protein was performed by 15 minutes incubation at 70 °C, which exploited *Sso*Pox extreme thermal stability [17,27,28]. Aggregated proteins were harvested by centrifugation (13 000 ×g, 25 °C, 30 min), and the estimated purity was evaluated as > 70%. The total protein quantity of samples was evaluated using a nanospectrophotometer (Nanodrop, Thermofisher Scientific; France). These protein amounts were used to calculate specific activities. This method enables fast comparison of variants’ activities, even if it might introduce a bias due to potential differences in expression/purification yields of the variants.

#### Phosphotriesterase activity screening

Phosphotriesterase activity was monitored using paraoxon (1 mM and 100 µM; Fig. **S1-1**) and CMP-coumarin (50 µM; methylphosphonic acid 3-cyano-4-methyl-2-oxo-2H-coumarin-7-yl ester cyclohexyl ester [37]; Fig. **S1-II**), a cyclosarin analog, as substrates. Kinetics experiments were performed in triplicate using 10 µL (for paraoxon hydrolysis) or 2 µL (for CMP-coumarin hydrolysis) of partially purified protein and recorded for 10 minutes. The time course of paraoxon (ε_405 nm_ = 17 000 M^-1^cm^-1^) and CMP-coumarin (ε_412 nm_ = 37 000 M^-1^cm^-1^) hydrolysis was performed at 25 °C and measured with a microplate reader (Synergy HT; BioTek, USA) and the Gen5.1 software in a 6.2 mm path length cell for a 200 µL reaction in a 96-well plate. Standard assays were performed in *pte buffer* (50 mM HEPES pH 8, 150 mM NaCl, 0.2 mM CoCl_2_ with pH adjusted at 25 °C using NaOH).

#### Lactonase activity screening

AHL lactonase activity has been screened using a genetically modified strain PAO1 of *P. aeruginosa* (JP2-pKD201) [38]. The pKD201 plasmid encodes proteins coding for bioluminescence production in the presence of 3-oxo-C12 AHLs in *P. aeruginosa*; the *las*I-*rhl*I genes, responsible of AHLs synthesis in wild-type *P. aeruginosa* [39], is deleted in the strain PAO1-JP2 (Fig. **S4**). *Sso*Pox variants (5 µL of tenfold diluted partially purified variants) are mixed in 100 µL of *pte buffer* with 3-oxo-C12 AHL (l) (100 nM; Fig. **S1-XII**) and incubated for 20 minutes at room temperature. A volume of 450 µL of LB media (Trimethoprime lactate 300 µg/mL to maintain the pKD201 plasmid) was inoculated by an overnight preculture of *P. aeruginosa* PAO1-JP2-pKD201 (1/50) and supplemented with the mixture protein/AHLs (50 µL). The final concentration of 3-oxo-C12 AHLs is 20 nM, prior to enzymatic hydrolysis by *Sso*Pox. After 270 minutes of culture at 37 °C, the cell density (OD_600 nm_) and bioluminescence (460-40 nm; intensity 100) from 200 µL aliquots of culture are measured in a 96-well plate using a microplate reader (Synergy HT, BioTek, USA) monitored using the Gen5.1 software. Controls in the same experiment are without enzyme and/or without AHLs.

### Purification of *SsoPox* and its variants for kinetic measurements and crystallographic studies

Protein production was performed using the *E. coli* strain BL21(DE_3_)-pGro7/GroEL (Takara Bio). Productions have been performed in 500 mL of ZYP medium [36] (100 µg/ml ampicillin, 34 µg/ml chloramphenicol) as previously explained [19,20,30], but 0.2% (w/v) arabinose (Sigma-Aldrich, France) was added to induce the expression of the chaperones GroEL/ES. Purification was performed as previously explained [30]. Briefly, a single step of 30 minutes incubation at 70 °C was performed, followed by differential ammonium sulfate precipitation, dialysis and exclusion size chromatography. Proteins were quantified using a nanospectrophotometer (Nanodrop, Thermofisher Scientific, France) and a protein molar extinction coefficient generated with the protein primary sequence in PROT-PARAM (Expasy Tool software) [40].

### Enzyme kinetics

Experiments were performed in triplicate at 25 °C and recorded using a microplate reader (Synergy HT, BioTek, USA) and the Gen5.1 software in a 6.2 mm path length cell for a 200 µL reaction in a 96-well plate as previously explained [19]. Catalytic parameters were obtained by fitting the data to the Michaelis-Menten (MM) equation [41] using the Graph-Pad Prism 5 software. When the V_max_ could not be reached in the experiments, the catalytic efficiency (k_cat_/K_M_) was obtained by fitting the linear part of MM plot to a linear regression using Graph-Pad Prism 5 software.

#### Phosphotriesterase kinetics

Phosphotriesterase activities have been determined as previously explained [19]. Briefly, standard assays were performed in *pte buffer* measuring the time course of hydrolysis of paraoxon (ε_405 nm_ = 17 000 M^-1^cm^-1^) and the nerve agent coumarin derivatives (CMP-coumarin, IMP-coumarin, PinP-coumarin) (Figure **S1-II-III-IV**) [37] (ε_412 nm_ = 37 000 M^-1^cm^-1^). Paraoxon and CMP-coumarin hydrolysis by *Sso*Pox-W263F were also evaluated in *pte buffer* supplemented with 0.1 and/or 0.01% SDS.

#### Lactonase kinetics

Lactonase kinetics were performed using a previously described protocol [11,19]. The time course hydrolysis of lactones were performed in *lac buffer* (2.5 mM Bicine pH 8.3, 150 mM NaCl, 0.2 mM CoCl_2_, 0.25 mM Cresol purple and 0.5% DMSO) over a concentration range 0-2 mM for AHLs and 0-5 mM for δ/γ-lactones. Time course hydrolysis of undecanoic-γ-lactone (*d*, l) (Figure **S1-XVI**) and 3-oxo-C10 AHLs (l) (Figure **S1-XI**) in presence of 0.1 and 0.01% SDS has also been performed in *lac buffer*. Duplicate kinetics of *Sso*Pox with 250 µM of racemic (*d*, l) and enantiopure (l) 3-oxo-C8 AHLs (Figure **S1-X**) have been performed to determine the enantioselectivity of *Sso*Pox. Cresol purple (pK_a_ 8.3 at 25 °C) is a pH indicator used to follow lactone ring hydrolysis by acidification of the medium. Its molar coefficient extinction (ε_577 nm_ = 2 923 M^-1^cm^-1^) was evaluated by recording the absorbance of the buffer over a range of acetic acid concentrations (0-0.35 mM). For some *Sso*Pox variants, the MM plots have been fitted to the substrate inhibition equation [41] using the Graph-Pad Prism 5 software to determine a K_I_ for undecanoic-δ-lactone. Consequently, the calculated catalytic efficiencies in these conditions are valid only at low substrate concentrations.

### Biophysical studies

#### Melting temperature determination

Circular Dichroism spectra were recorded as previously explained [19] using a Jasco J-810 spectropolarimeter equipped with a Pelletier type temperature control system (Jasco PTC-4235) in a 1 mm thick quartz cell and using the Spectra Manager software. Briefly, measurements were performed in 10 mM sodium phosphate buffer at pH 8 with a protein concentration of 0.1 mg/mL. Denaturation was recorded at 222 nm by increasing the temperature from 20 to 95 °C (at 5 °C/min) in 10 mM sodium phosphate buffer at pH 8 containing increasing concentrations (1.5-4 M) of guanidinium chloride. The theoretical T_m_ without guanidinium chloride was extrapolated by a linear fit using the GraphPadPrism 5 software.

#### Thermophilicity analysis

The temperature dependence of the paraoxonase activity of *Sso*Pox variants were studied over the range of temperatures 25-85 °C with a 10 °C increment. The paraoxon hydrolysis (50 µM) (ε_405 nm_ = 17 000 M^-1^cm^-1^) was monitored in 500 µL with 1-cm path length cell and a Cary WinUV spectophotometer (Varian, Australia) using the Cary WinUV software in *pte buffer* pH adjusted with NaOH to pH 8 at each temperature. Measurements were performed in triplicate.

### Structural analysis

#### Crystallization

Crystallization assays were performed as previously described [30,42] using enzymes concentrated at 6 mg/mL^-1^. Co-crystallization assays with C10-HTL were performed using the same protocol [22] but adding 4 µL of a 60 mM C10-HTL solution (in Ethyl acetate:DMSO; 1:1) to 120 µL of the protein solution. Crystallization was performed using the hanging drop vapor diffusion method in 96 well plates (Greiner Microplate, 96 well, PS, F-bottom) on ViewDrop II seals (TPP Labtech). Equal volumes (0.5 µL) of protein and reservoir solutions were mixed using a HoneyBee X-8 (Cartesian) crystallization instrument, and the resulting drops were equilibrated against a 150 µL reservoir solution containing 20-30% (w/v) PEG 8000 and 50 mM Tris-HCl buffer (pH 8). Thin crystals appeared after few days at 277 K.

#### Data collection and structure determination

Crystals were first transferred to a cryoprotectant solution composed of the reservoir solution and 20% (v/v) glycerol, a 1/30 (v/v) ratio of a 60 mM C10 HTL solution was added to the cryo-protectant solution for co-crystallization trials. Crystals were then flash-cooled in liquid nitrogen. X-ray diffraction data were collected for W263L, W263M, W263I, and W263V crystals at 100 K using synchrotron radiation at the ID23-1 beam line (ESRF, Grenoble, France) and an ADSC Q315r detector. The diffraction data for W263F and W263I-C10 HTL crystals were collected at the Proxima-1 beam line (SOLEIL, Gif-sur-Yvette, France) using a PILATUS-6M detector. X-ray diffraction data were integrated and scaled with the *XDS* package [43] (Table **4**). The phases were obtained using the native structure of *Sso*Pox (PDB code 2vc5) as a starting model, performing a molecular replacement with *MOLREP* [44] or *PHASER* [45]. The models were built with *Coot* [46] and refined using *REFMAC* [47]. Structure illustrations were performed using *PyMOL* [48]. B-averages of structures were evaluated using B-average software from CCP4 suite [49]. RMSD were evaluated comparing all structures to wild-type structure (2vc5) using Swiss Prot PDB Viewer software [50].

**Table 4:** Data collection and refinement statistics of *Sso*Pox variants structures.

Data collections	**W263T**	**W263V**	**W263I**	**W263M**	**W263L**	**W263F**	**W263I-HTL**
PDB ID	4KES	4KER	4KET	4KEU	4KEV	4KEZ	4KF1
Beamline	ID23-EH1	ID23-EH1	ID23-EH1	ID23-EH1	ID23-EH1	Proxima-1	Proxima-1
Wavelength (A°)	0.99987	0.99987	0.99987	0.99987	0.99987	1.00882	0.954
Resolution (A°) (last bin)	2.1	2.6	2.0	2.2	2.65	1.85	2.0
Space group	P2_1_2_1_2_1_						
**Unit cell dimensions**							
a (A°)	84.2	87.2	87.4	86.8	86.60	87.13	86.78
b (A°)	103.6	103.50	103.9	103.9	105.00	103.62	103.53
c (A°)	151.8	151.60	150.5	151.6	153.60	151.66	151.71
No. observed reflections	501100 (63243)	301273 (30897)	552123 (67740)	566547 (70185)	256359 (39151)	878146 (68071)	688907 (93622)
No. unique reflections	78058 (10044)	42787 (4506)	92731 (12507)	70219 (8674)	39816 (5986)	117479 (8927)	92509 (12379)
Completeness (%)	99.9 (100)	99.7 (99.6)	99.6 (99.8)	99.9 (99.9)	99.5 (99.8)	100 (100)	99.6 (99.1)
Rsym (%)	8.6 (37.6)	12.5 (45.3)	10.7 (37.9)	11.1 (44.6)	10.2 (54.5)	7.1 (47.1)	10.1 (45.9)
Rmeasure (%)	8.2 (48.9)	11.9 (55.4)	10.4 (44.2)	10.6 (55.8)	7.3 (54.5)	7.7 (50.5)	10.8 (49.2)
I/σ(I)	16.43 (4.66)	16.46 (4.40)	13.19 (4.15)	15.78 (4.59)	19.31 (4.17)	18.28 (4.58)	15.05 (4.79)
Last resolution shell	2.2 - 2.1	2.7 - 2.6	2.1 - 2.0	2.3 - 2.2	2.8 - 2.65	1.9 - 1.85	2.1 - 2.0
Redundancy	6.41 (6.30)	7.04 (6.86)	5.95 (5.42)	8.07 (8.09)	6.44 (6.54)	7.47 (7.63)	7.45 (7.56)
**Refinement statistics**							
Resolution range (last bin) (A°)	49.52 - 2.1 (2.154 - 2.100)	45.41 - 2.6 (2.667 - 2.600)	44.66 - 2.0 (2.052 - 2.000)	49.14 - 2.2 (2.257 - 2.200)	86.776 - 2.652 (2.721 - 2.652)	49.03 - 1.85 (1.898 - 1.850)	45.44 - 2.0 (2.052 - 2.000)
No. Reflections	74154	40646	88093	66708	37816	111605	121721
Rwork (last bin) (%)	16.26 (19.8)	16.83 (22.5)	14.47 (16.8)	17.02 (21.6)	20.30 (32.2)	15.13 (18.9)	14.75 (18.0)
Rfree (last bin) (%)	20.54 (23.0)	23.74 (31.1)	18.95 (22.9)	21.53 (27.0)	26.70 (40.3)	18.64 (22.5)	18.81 (23.0)
No. protein atoms	10280	10146	10263	10225	10058	10151	10346
No. water molecules	579	365	900	332	132	1049	1042
Average B factor (A°^2^)	38.74	36 .60	24.11	35.62	75.95	25.67	22.24
**RMSD from ideal**							
Bond lengths (A°)	0.0041	0.0172	0.0052	0.0216	0.0146	0.0040	0.0081
Bond angles (°)	0.8481	0.5669	0.9531	0.6745	0.5081	0.8958	0.6012

## Supporting Information

Figure S1 **Chemical structure of OPs (I-IV), AHLs (V-XII), γ-lactones (XIII-XVII), δ-lactones (XVIII-XXI) and other lactones (XXII-XIII).**
(DOCX)

Figure S2 **Enantiopreference of wild-type *Sso*Pox for AHLs.**
(DOCX)

Figure S3 **Supplemental *Sso*Pox-W263 saturation site OP hydrolase activity screening.**
(DOCX)

Figure S4 **Schematic representation of *P. aeruginosa* based AHLase screening method.**
(DOCX)

Figure S5 **Further catalytic efficiency comparisons between selected variants.**
(DOCX)

Figure S6 **Structural comparisons of selected variants and *wt Sso*Pox.**
(DOCX)

Figure S7 **Active site cavity representation of wild-type *Sso*Pox and all selected variants.**
(DOCX)

Figure S8 **Structural analysis of *Sso*Pox-W263I HTL bound structure.**
(DOCX)

Figure S9 **RMSD and B-average comparison of wild-type *Sso*Pox and its variants.**
(DOCX)

Table S1 **Biophysics parameters of *wt Sso*Pox and its variants.**
(DOCX)
