# Supplementary material for: Differential Active Site Loop Conformations Mediate Promiscuous Activities in the Lactonase SsoPox
Source: PLoS One. 2013 Sep 23;8(9):e75272. doi: 10.1371/journal.pone.0075272 (PMC3781021; doi:10.1371/journal.pone.0075272)
Supplement: Figure S1 — Chemical structure of OPs (I-IV), AHLs (V-XII), γ-lactones (XIII-XVII), δ-lactones (XVIII-XXI) and other lactones (XXII-XIII). (DOCX) [file pone.0075272.s001.docx]

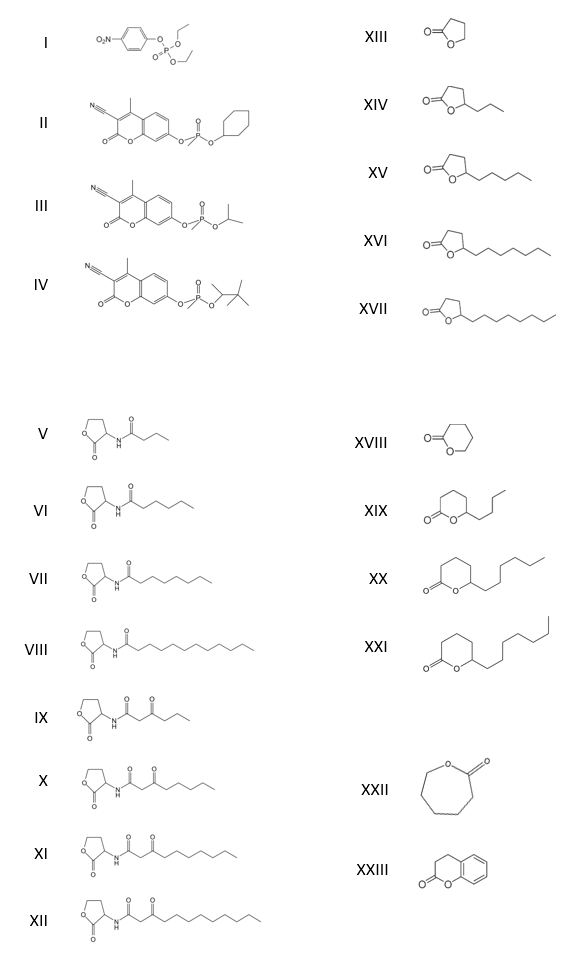


**Figure S1: Chemical structure of OPs (I-IV), AHLs (V-XII), γ-lactones (XIII-XVII), δ-lactones (XVIII-XXI) and other lactones (XXII-XIII)**

Chemical structures of (**I**) Ethyl-Paraoxon, (**II**) CMP-coumarin, (III) IMP-coumarin, (IV) PinP-coumarin [[1](#_ENREF_1)], C4-AHL (**V**), C6-AHL (**VI**), C8-AHL (**VII**), C12-AHL (**VIII**), 3-oxo-C6 AHL (**IX**), 3-oxo-C8 AHL (**X**), 3-oxo-C10 AHL (**XI**), 3-oxo-C12 AHL (**XII**), γ-butyrolactone (**XIII**), γ-heptanolide (**XIV**), Nonanoic-γ-lactone (**XV**), Undecanoic-γ-lactone (**XVI**), Dodecanoic-γ-lactone (**XVII**), δ-valerolactone (**XVIII**), Nonanoic-δ-lactone (**XIX**), Undecanoic-δ-lactone (**XX**), Dodecanoic-δ-lactone (**XXI**), ε-caprolactone (**XXII**) and Dihydrocoumarin (**XXIII**) are presented.

1. Ashani Y, Gupta RD, Goldsmith M, Silman I, Sussman JL, et al. (2010) Stereo-specific synthesis of analogs of nerve agents and their utilization for selection and characterization of paraoxonase (PON1) catalytic scavengers. Chem Biol Interact 187: 362-369.
