# Supplementary material for: Differential Active Site Loop Conformations Mediate Promiscuous Activities in the Lactonase SsoPox
Source: PLoS One. 2013 Sep 23;8(9):e75272. doi: 10.1371/journal.pone.0075272 (PMC3781021; doi:10.1371/journal.pone.0075272)
Supplement: Figure S2 — Enantiopreference of wild-type SsoPox for AHLs. (DOCX) [file pone.0075272.s002.docx]

**
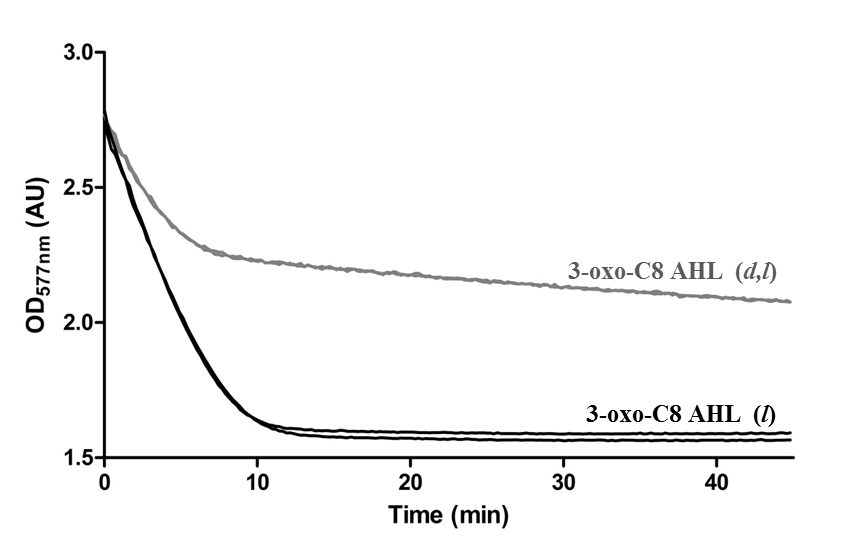
**

**Figure S2: Enantiopreference of wild-type *Sso*Pox for AHLs**

Time course hydrolysis of racemic (grey) and pure levorotatory (black) 3-oxo-C8 AHLs (250 µM) by *Sso*Pox is followed in duplicate at 25 °C and at 577 nm. See methods for more details.
