# Supplementary material for: Differential Active Site Loop Conformations Mediate Promiscuous Activities in the Lactonase SsoPox
Source: PLoS One. 2013 Sep 23;8(9):e75272. doi: 10.1371/journal.pone.0075272 (PMC3781021; doi:10.1371/journal.pone.0075272)
Supplement: Figure S3 — Supplemental SsoPox-W263 saturation site OP hydrolase activity screening. (DOCX) [file pone.0075272.s003.docx]

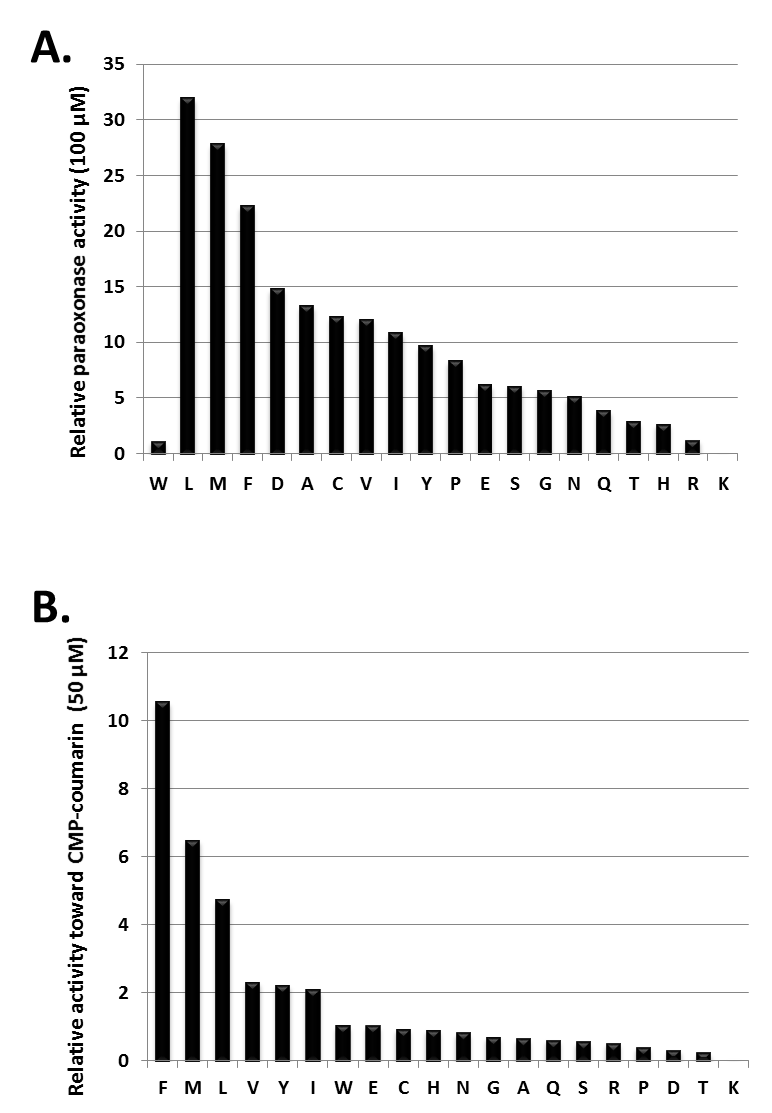


**Figure S3: Supplemental *Sso*Pox-W263 saturation site OP hydrolase activity screening**

Relative activities of W263 saturation site variants have been screened with 100 µM (**A.**) of paraoxon substrate and 50 µM (**C**.) of CMP-coumarin substrate.
