# Supplementary figures and images for: Differential Active Site Loop Conformations Mediate Promiscuous Activities in the Lactonase SsoPox
Source: PLoS One. 2013 Sep 23;8(9):e75272. doi: 10.1371/journal.pone.0075272 (PMC3781021; doi:10.1371/journal.pone.0075272)

**
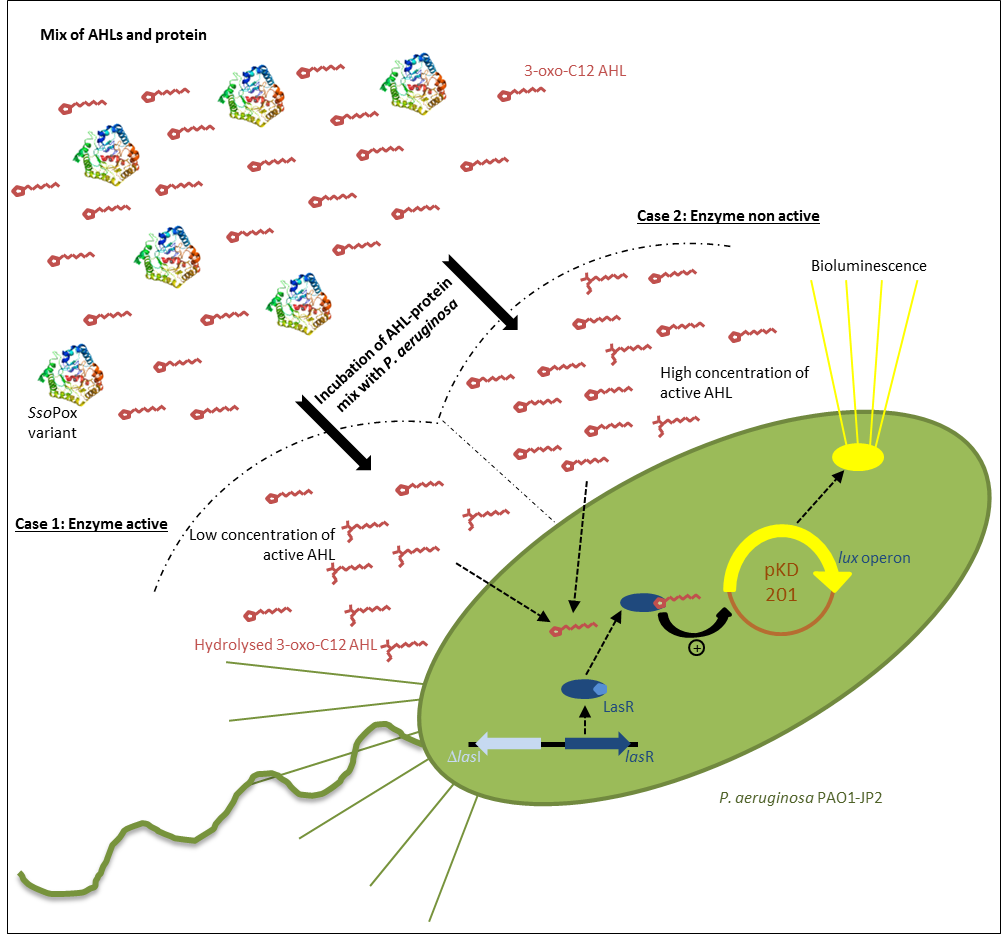
**

**Figure S4: Schematic representation of *P. aeruginosa* based AHLase screening method**

Supplement: Figure S4 — Schematic representation of P. aeruginosa based AHLase screening method. (DOCX) [file pone.0075272.s004.docx]

**
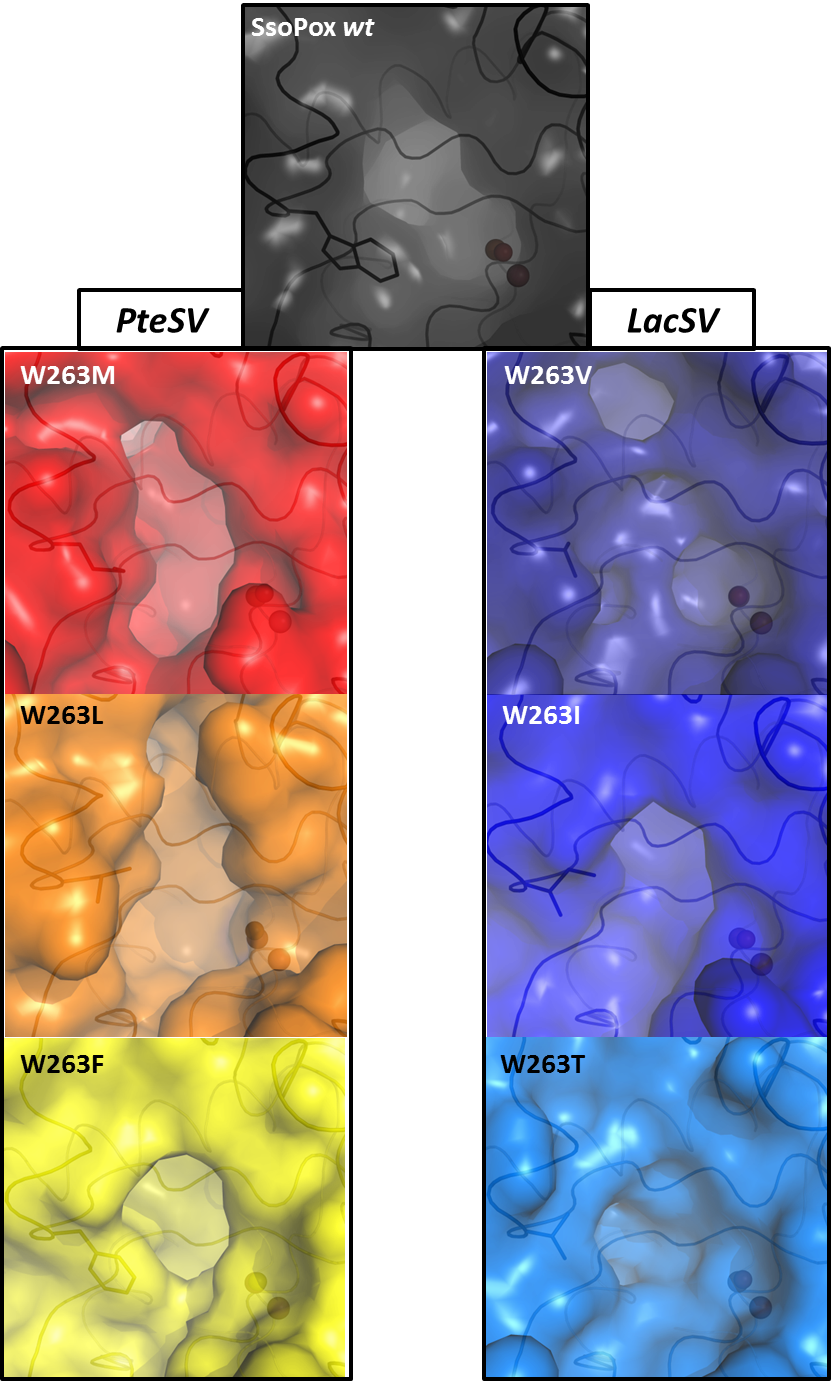
**

**Figure S7: Active site cavity representation of wild-type *Sso*Pox and all selected variants**

Supplement: Figure S7 — Active site cavity representation of wild-type SsoPox and all selected variants. (DOCX) [file pone.0075272.s007.docx]
