# Supplementary material for: Differential Active Site Loop Conformations Mediate Promiscuous Activities in the Lactonase SsoPox
Source: PLoS One. 2013 Sep 23;8(9):e75272. doi: 10.1371/journal.pone.0075272 (PMC3781021; doi:10.1371/journal.pone.0075272)
Supplement: Figure S5 — Further catalytic efficiency comparisons between selected variants. (DOCX) [file pone.0075272.s005.docx]

**
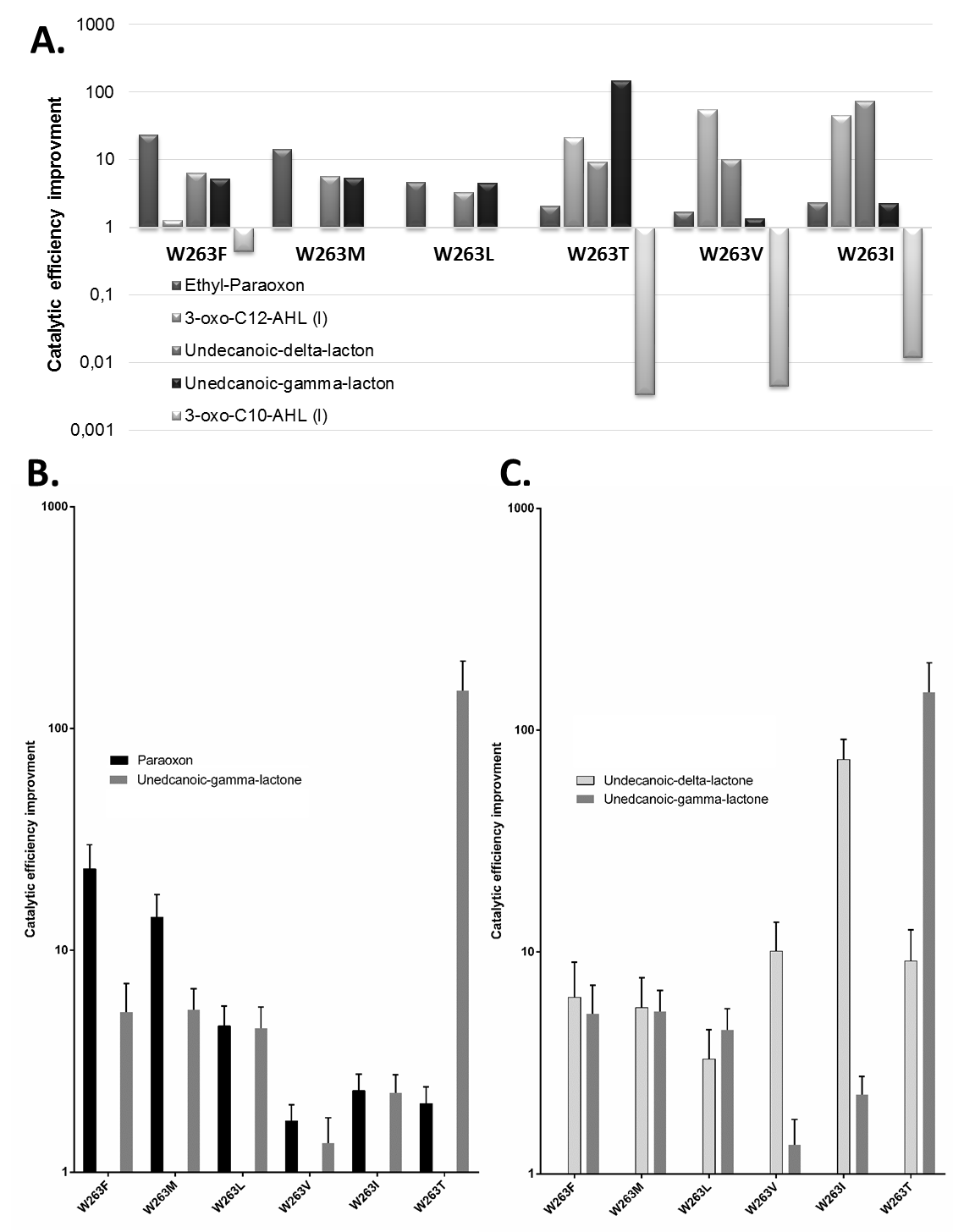
**

**Figure S5: Further catalytic efficiency comparisons between selected variants**

Catalytic efficiency comparisons of variants for paraoxon (**A. & B.**), undecanoic-γ-lactones (**A. B. & C.**), undecanoic-δ-lactones (**A. & C.**), 3-oxo-C10 AHLs (**A.**) and 3-oxo-C10 AHLs (**A.**).
