# Supplementary material for: Differential Active Site Loop Conformations Mediate Promiscuous Activities in the Lactonase SsoPox
Source: PLoS One. 2013 Sep 23;8(9):e75272. doi: 10.1371/journal.pone.0075272 (PMC3781021; doi:10.1371/journal.pone.0075272)
Supplement: Figure S6 — Structural comparisons of selected variants and wt SsoPox. (DOCX) [file pone.0075272.s006.docx]

**
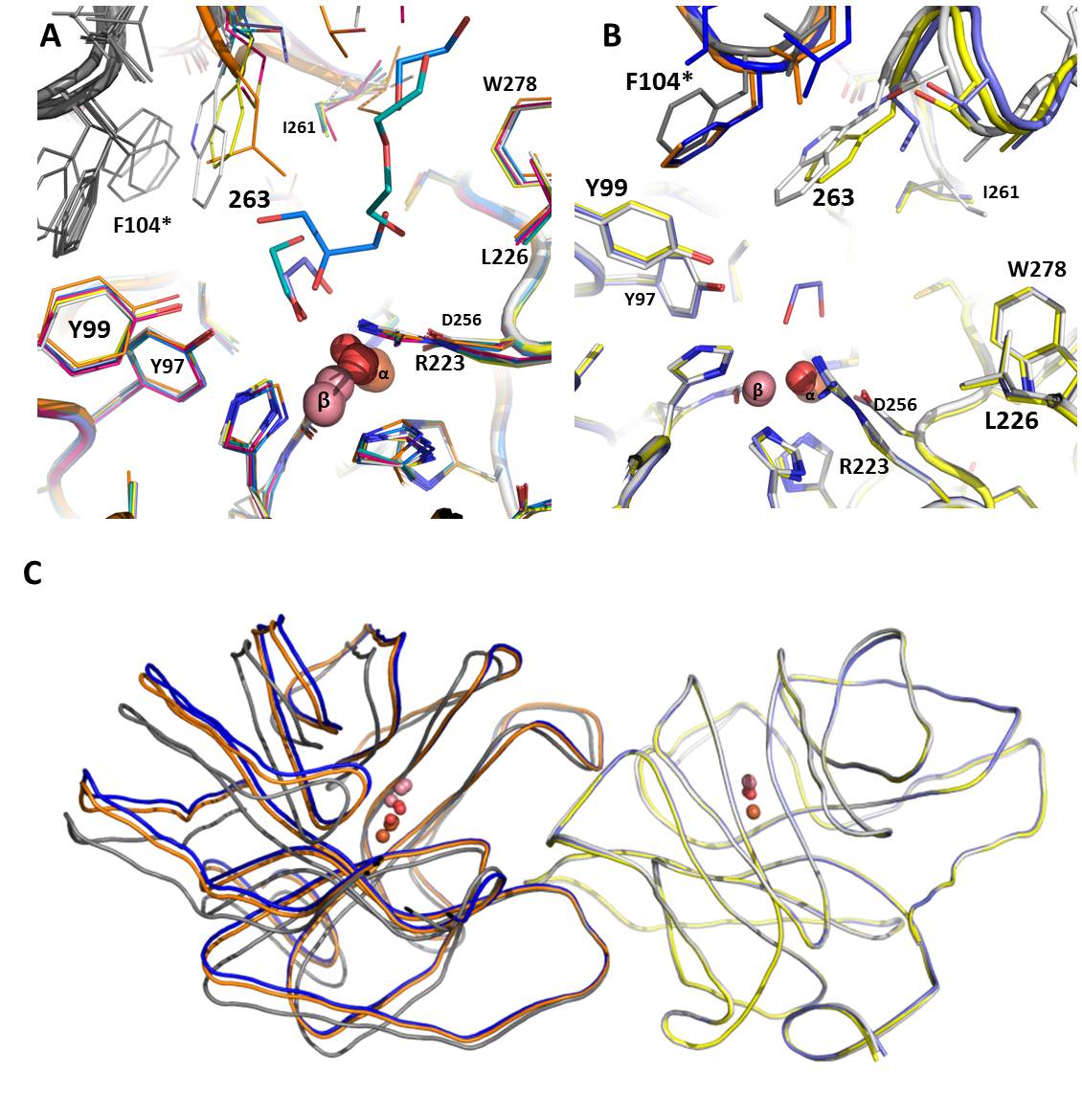
**

**Figure S6: Structural comparisons of selected variants and *wt Sso*Pox**

**A.** Structural superposition of selected variants (V: blue; T: light blue; I: light violet; F: yellow; L: orange; M: pink) and *wt Sso*Pox (light grey) at the active site region. The second monomer of each structure is represented in dark grey; the F104 of this second monomer is indicated with a star. **B.** Structural superposition of representing member of *PteSV* (*Sso*Pox-W263F; first monomer: yellow; second monomer: orange), *LacSV* (*Sso*Pox-W263I; first monomer: light violet; second monomer: dark blue) and *wt Sso*Pox (first monomer: light grey; second monomer: dark grey) at the active site region. The F104 of the second monomer is indicated with a star. **C.** Structural comparison of dimer compaction with structural superposition of first monomer and observing impact on the second monomer of *wt Sso*Pox, W263F and W263I (colour codes are the same as before).
