# Supplementary material for: Differential Active Site Loop Conformations Mediate Promiscuous Activities in the Lactonase SsoPox
Source: PLoS One. 2013 Sep 23;8(9):e75272. doi: 10.1371/journal.pone.0075272 (PMC3781021; doi:10.1371/journal.pone.0075272)
Supplement: Figure S8 — Structural analysis of SsoPox-W263I HTL bound structure. (DOCX) [file pone.0075272.s008.docx]

**
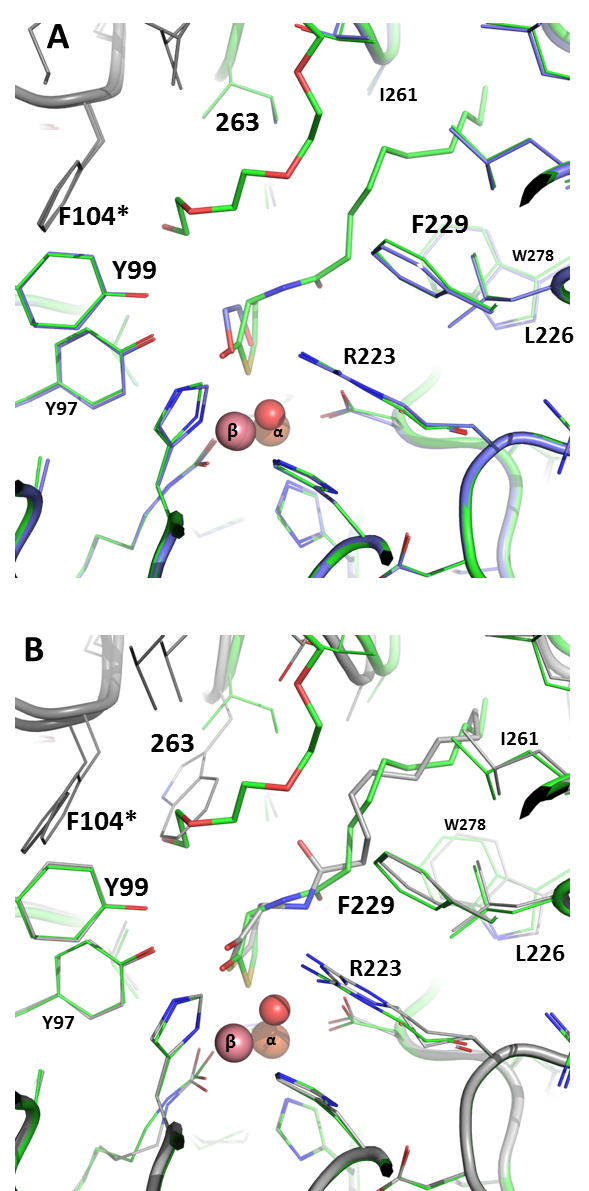
**

**Figure S8: Structural analysis of *Sso*Pox-W263I HTL bound structure**

**A.** Structural superposition of apo (light violet) and HTL-bound (green) structures of *Sso*Pox-W263I in the active site region. **B.** Structural superposition of HTL-bound structures of *wt Sso*Pox (light grey) and *Sso*Pox-W263I (green) at the active site region. The F104 residue indicated with a star corresponds to a residue of the second monomer of the dimer which is dark grey for all structures.
