# Supplementary material for: Differential Active Site Loop Conformations Mediate Promiscuous Activities in the Lactonase SsoPox
Source: PLoS One. 2013 Sep 23;8(9):e75272. doi: 10.1371/journal.pone.0075272 (PMC3781021; doi:10.1371/journal.pone.0075272)
Supplement: Figure S9 — RMSD and B-average comparison of wild-type SsoPox and its variants. (DOCX) [file pone.0075272.s009.docx]

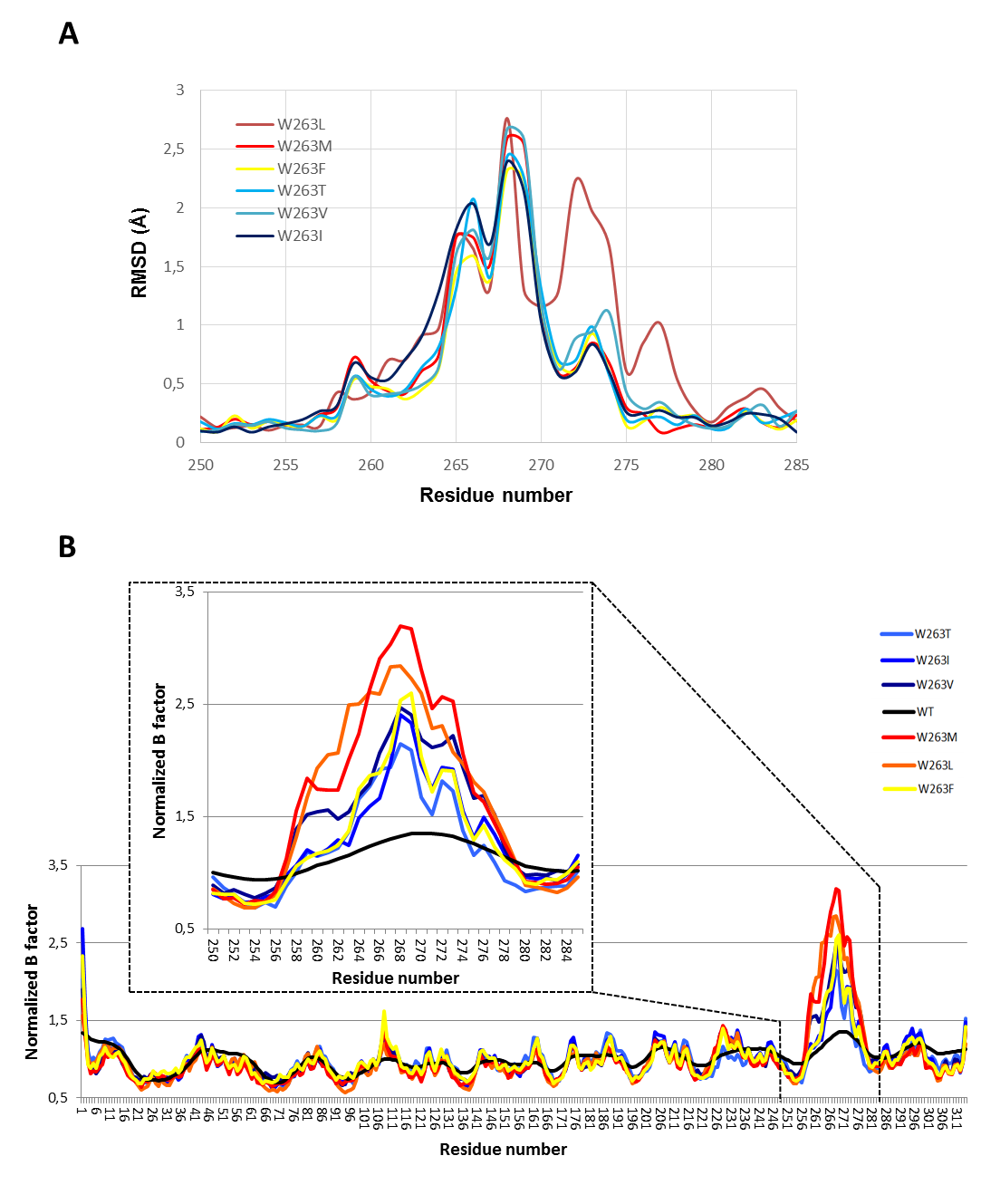


**Figure S9: RMSD and B-average comparison of wild-type *Sso*Pox and its variants**

**A.** RMSD analysis of the selected variants compared to wild-type enzyme at the amino acids 250-285. **B.** B-factor analysis along the backbone chain of all selected variants and wild-type *Sso*Pox. Normalized B-factors are represented; it corresponds to the mean, on the four molecules by asymmetric unit, of B-factor of each residues normalized by the mean B-factor of the structure.
