# Supplementary material for: Differential Active Site Loop Conformations Mediate Promiscuous Activities in the Lactonase SsoPox
Source: PLoS One. 2013 Sep 23;8(9):e75272. doi: 10.1371/journal.pone.0075272 (PMC3781021; doi:10.1371/journal.pone.0075272)
Supplement: Table S1 — Biophysics parameters of wt SsoPox and its variants. (DOCX) [file pone.0075272.s010.docx]

**Table SI: Biophysics parameters of *wt* *Sso*Pox and its variants**

| ***Sso*Pox** | *wt*^#^ | W263F | W263M | W263L | W263T | W263V | W263I |
| --- | --- | --- | --- | --- | --- | --- | --- |
| **Melting temperature (°C)** | 104 | 91.8 ± 1.7 | 85.3 ± 0.9 | 92.0 ± 2.1 | 89.2 ± 0.4 | 84.1 ± 1.6 | 87.8 ± 1.2 |
| **Normalized B factor (Pos 250 to 280)** | 1.145 ± 0.051 | 1.366 ± 0.038 | 1.766 ± 0.073 | 1.690 ± 0.253 | 1.266 ± 0.146 | 1.627 ± 0.057 | 1.342 ± 0.028 |
| **Relative movement compaction of dimers compared to *wt Sso*Pox (Å)^§^** | - | 3.8 | 3.7 | -1.3 | 3.4 | 3.8 | 4.7 |

* The paraoxonase thermophilicity has been evaluated from data represented on supplemental figure 2.

# Data obtained are from Merone *et al*. (2005) for thermophilicity, Elias *et al*. (2008) for B factor and Del Vecchio *et al*. (2009) for Tm.

§ The values correspond to the relative movement between the variants and *wt Sso*Pox monomers while the second monomers implicated in dimers have been superposed. It relates the higher compaction (positive value) or relaxation (negative value) of the dimer as compared to *wt Sso*Pox.
